# Valorization of Lignin and Its Derived Molecules by Electrocatalytic Oxidation

**DOI:** 10.1021/acssuschemeng.5c02675

**Published:** 2025-09-25

**Authors:** Parminder Kaur, Jiaqi Wang, Xiang Li, Reetta Karinen, Georgia Papanikolaou, Paola Lanzafame, Gabriele Centi, Yongdan Li

**Affiliations:** † Geological Survey of Finland, P.O. Box 96, FI-02151 Espoo, Finland; ‡ Department of Chemical and Metallurgical Engineering, 174277 Aalto University, 00076 Espoo, Finland; § Department ChiBiofarAm, University of Messina, Italy and ERIC Aisbl (European Research Institue of Catalysis), 98122 Brussels, Belgium

**Keywords:** electrocatalytic oxidation, lignin molecules, laccase mediator system, electrolytic mediated system

## Abstract

Lignin, an abundant biopolymer within the biosphere, represents a promising renewable source of organic chemicals and fuels. Within the framework of a sustainable biorefinery, efficient lignin valorization plays a pivotal role in enhancing the economic feasibility of a holistic biomass transformation. Electrocatalytic lignin oxidation (E-LignoX) emerges as an innovative strategy to upgrade lignin into high-value bioproducts, offering an economically viable and environmentally benign alternative seamlessly adaptable to the existing biorefinery infrastructures. With recent advances in electrode design, mediator systems, and process optimization, E-LignoX stands at the forefront of innovative lignin valorization strategies. This perspective explores the transformative potential of E-LignoX, emphasizing its capacity to seamlessly integrate into the existing biorefinery frameworks while offering a scalable, cost-effective alternative to conventional catalytic methods. Key considerations include (i) the diversity of lignin-derived molecules suitable for electrocatalytic upgrading, (ii) the design and advancement of high-performance anodic electrodes, (iii) the role of mediators in enhancing process efficiency, and (iv) a comparative outlook on direct versus mediated E-LignoX pathways. By critically assessing the advantages and challenges of this emerging technology, we highlight its role in reshaping the sustainable production of biofuels and biochemicals, ultimately paving the way for a circular and fossil-independent bioeconomy.

## Graphical abstract

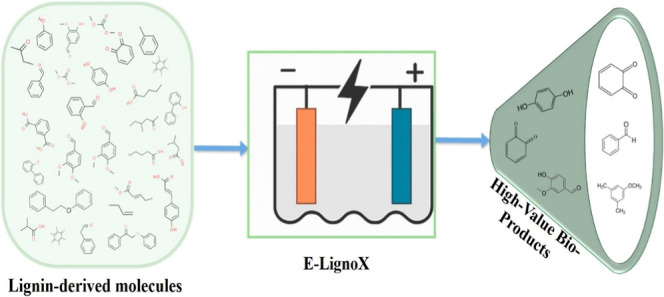

## Introduction

The growing global population’s rising fossil fuel demand drives the urgent need for alternative energy and chemical sources.^1^ Biomass, especially lignin, a complex, recalcitrant phenolic polymer constituting 20–35% of dry lignocellulose and derived from phenylpropane units (e.g., coniferyl and sinapyl alcohols), is a promising renewable resource.^2^ However, industries extract 50 million tons of lignin annually, yet only 2% is used in low-value applications (e.g., dispersion or binding agents), with the remainder burned for energy.^3^ Similarly, lignocellulosic biorefineries primarily incinerate lignin to generate heat and power, for example, a typical bioethanol plant produces about 70,000 tons of lignin per year from corn stover.^4^ Currently, roughly 95% of lignin is burned, yielding a value of about $150 per ton, whereas complete conversion into aromatics could boost its value to around $1200 per ton.^5^ Lignin’s complex, recalcitrant nature necessitates pretreatment or fractionation much like oil refineries to enable its conversion into a broad range of biobased products (Figure 1). Overcoming the technical, economic, and reactivity challenges associated with lignin valorization requires innovative strategies.^6,7^

**Figure 1:**
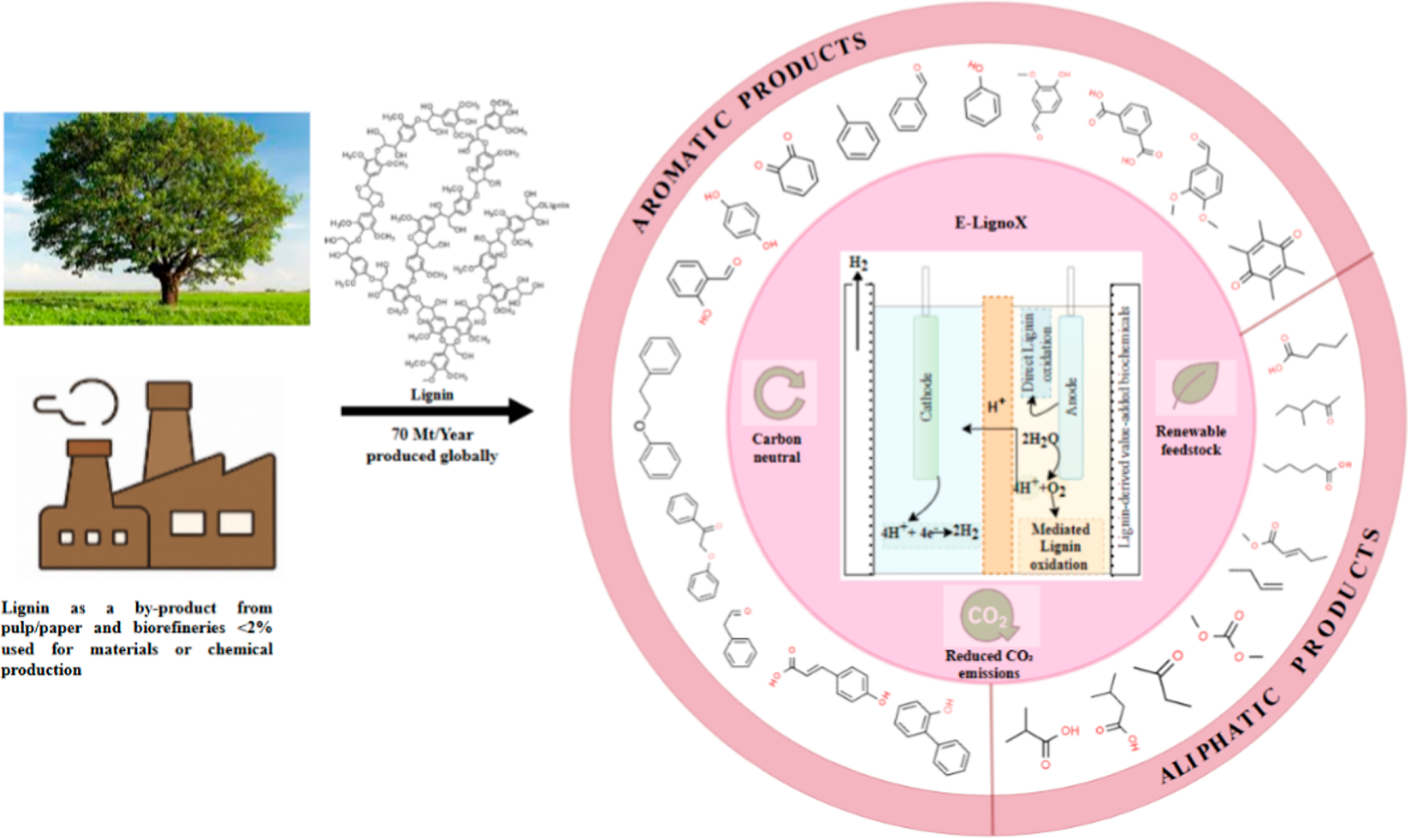
Sustainable valorization of lignin via electrocatalytic oxidation (E-LignoX).

Lignin depolymerization can be achieved by several methods. Reductive strategies use metal catalysts (e.g., Cu-doped oxides and noble metals) for bond cleavage via hydrogenation, hydrogenolysis, and hydrodeoxygenation;^8–10^ solvolysis leverages solvents (water and alcohols) with metals to boost bio-oil yields;^11–13^ pyrolysis thermally breaks weak bonds at moderate temperatures (producing vanillin/syringol) but risks coke formation at very high temperatures;^14–16^ oxidative methods target β-O-4 linkages using H_2_O_2_/catalysts, though overoxidation persists;^17–19^ and biological approaches utilize enzymes/microbes under mild conditions, limited by scalability.^20–22^ Lignin-derived molecules are a more practical feedstock for biofuels, biochemicals, and biomaterials than lignin. Lignin requires complex reactions to break its structure, whereas lignin monomers with their specific functional groups allow for simpler, more efficient synthesis and precise customization.^23–25^ Advances in innovative technologies are further unlocking lignin’s potential as a valuable, renewable resource.

Recent research has focused on electrochemical lignin valorization, primarily via oxidation to produce aromatic monomers and dimers for biobased chemicals and fuels.^26^ E-LignoX uses applied potential and tailored electrodes to cleave lignin linkages, especially β-O-4 bonds, enhancing hydroxyl and carboxyl functionalities and yielding compounds like aromatic aldehydes and acids.^27–29^ E-LignoX benefits from renewable energy use, reducing fossil fuel reliance and carbon footprint, and it can be paired with cathodic processes for tandem reactions that improve efficiency.^30–33^ However, high kinetic barriers and the presence of reactive radical species near the electrode present significant challenges; nonetheless, mediators can address these issues via heterogeneous electron transfer.^34^ Besides, anode materials also facilitate electron transfer during lignin oxidation. Studies on metals, metal oxides, and carbon-based materials reveal that these electrodes influence selectivity and productivity by directing electrons to specific functional groups while requiring robust long-term stability.^35–37^ Ongoing research aims to enhance their electrocatalytic activity, durability, and selectivity, underscoring the need for innovative anode development to advance organic electrosynthesis.

The perspective explores the potential of lignin-derived molecules in oxidation reactions, emphasizing their role in generating high-value bioproducts. Some sustainable applications in biofuel production and biochemical synthesis are exemplified, leveraging lignocellulosic biomass and industrial byproducts to enhance the economic feasibility of biorefineries. A section on lignin monomers and derivatives as feedstocks is presented, focusing on achieving a higher selectivity in producing industrial-grade chemicals and materials. By highlighting the promising role of E-LignoX, this perspective underscores its potential for synthesizing valuable bioproducts while summarizing the advantages and challenges of direct and mediated E-LignoX routes, emphasizing anodic electrode design. Finally, the multifaceted benefits of integrating E-LignoX into lignin valorization are discussed, suggesting that continued research and development could drive significant advancements toward more efficient and environmentally friendly biochemical production.

## Lignin-Derived Molecules

Lignin depolymerization yields diverse molecules (monomers, dimers, and oligomers) influenced by botanical origin and extraction methods (Figure 2).^27,36,38–41^ A bibliographic review identified key aromatic compounds from lignin depolymerization (Table 1) that often undergo oxidation to form industrially valuable chemicals, with selectivity depending on the catalytic systems used.^42,43^ For example, phenol oxidizes to catechol/hydroquinone; guaiacol to vanillin/syringaldehyde; and benzyl alcohol to benzaldehyde. Vanillin, a key food flavoring, arises from the β-O-4 linkage cleavage in lignin. Similarly, toluene and xylenes yield benzaldehyde/benzoic acid, while styrene oxidation produces benzaldehyde, vanillin, and benzoquinone. These molecules serve as versatile precursors for flavors, fragrances, and pharmaceuticals.

**Figure 2:**
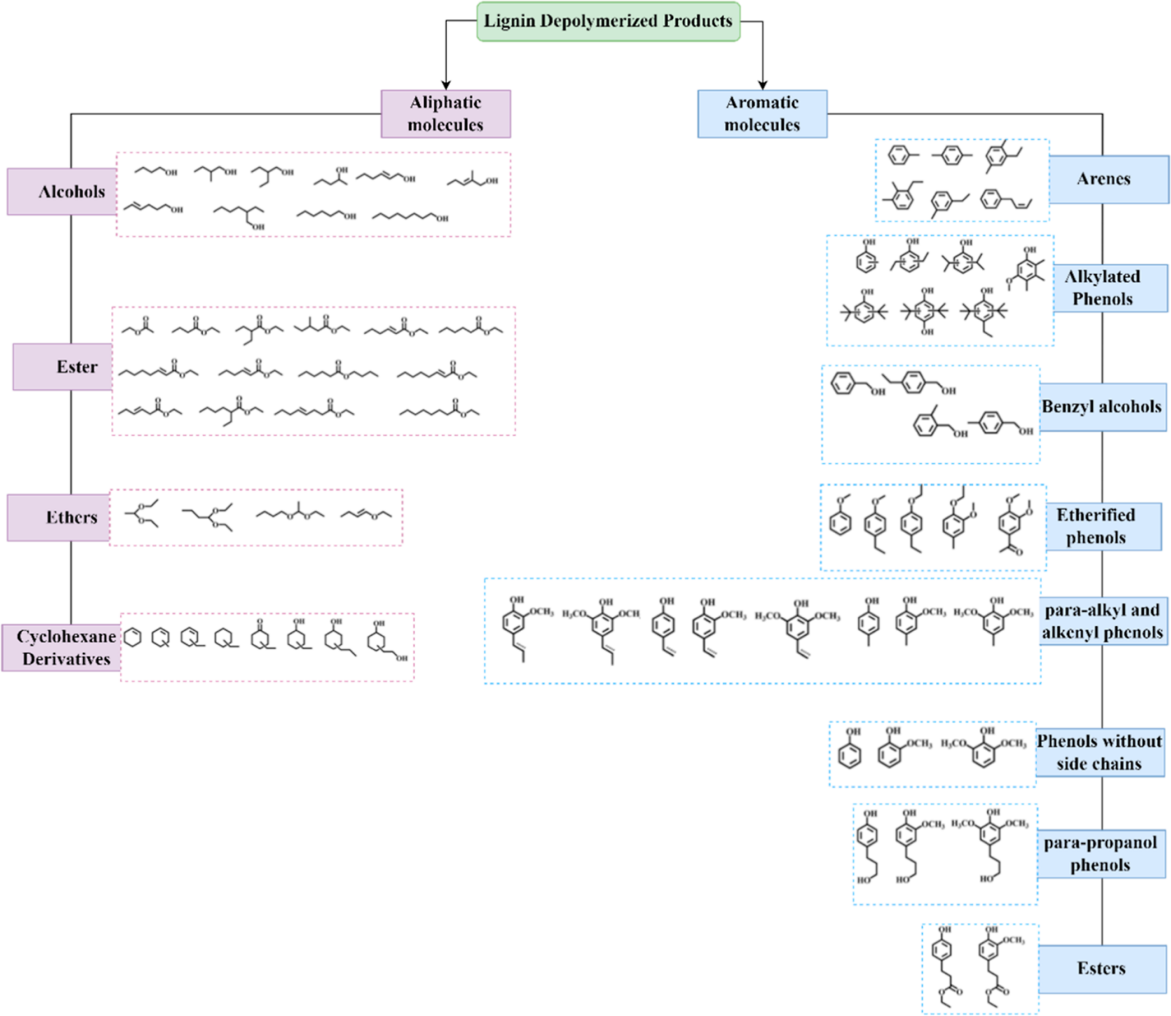
Classification of lignin-depolymerized molecules. Adapted with permission from ref 38. Copyright 2023 Elsevier.

**Table 1:** Lignin-Derived Aromatic Molecules: Properties, Oxidation Potential, Products, and Uses of Oxidized Products

s. no	common name	molecular formula	molecular weight g/mol	density Kg/m^3^ (298 K)	solubility in water (g/L)	pH	oxidation potential (V)	oxidation products	use of product	references
1	phenol	C_6_H_6_O	94.11	1065	83	8–12	+0.65 V	hydro quinone	used as antioxidants and reducing agents in the photography/rubber industry	^56,57^
2	catechol	C_6_H_6_O_2_	110	1344	430	<7	+0.20 V	1,2-benzoquinone	use in hair coloring products and azo dyes.	^58^
3	resorcinol	C_6_H_6_O_2_	110.11	1280	1100	9.14	+0.60 V	dimer product	used in adhesives and resins; also serves as an essential building block for drug development.	^59^
4	guaiacol	C_7_H_8_O_2_	124.13	1112	23.3	5.4	+1.6 V	3,3′-dimethoxy-4,4′-biphenol, 1,3-benzodioxole, and ortho-quinone	rubber industry for curing of elastomers	^60,61^
5	vanillin	C_8_H_8_O_3_	152.14	1056	30–50	7.78	+1.8 V	2-methoxy hydroquinone	use as an electrolyte in aqueous redox flow	^62,63^
6	benzyl alcohol	C_7_H_6_O	108.14	980	40	7	+1.1	benzaldehyde	use in cosmetics as a denaturant, a flavoring agent, and as a fragrance	^64^
7	toluene	C_7_H_8_	92.1	862	0.52		+2.26	benzaldehyde and methyl anisole	used in the flavor and fragrance industry, pharmaceutical industry, perfumes and cosmetics	^65,66^
8	p-xylene	C_8_H_10_	106.17	849	0.156		+2.01	dimethyl anisole	used as a solvent and in the synthesis of other chemicals and dyes	^66^
9	m-xylene	C_8_H_10_	106.17	860	0.146		∼ + 2	isophthalic acid	used to produce coatings, polyester resins, unsaturated polyester resins	^67^
10	o-xylene	C_8_H_10_	106.17	879	0.171		+0.90	phthalic anhydride	use in producing polyester resins, paints and lacquers, insect repellents, urethane polyester polyols, rubber scorch inhibitors, and retarders.	^68^
11	anisole	C_7_H_8_O	108.13	995	1.6		+1.76	benzoquinone	fungicide, as a reagent in photography, and to prepare dyes and other chemicals	^69^
12	benzene	C_6_H_6_	78.11	876	1.79	4	+2.48	phenol	used in the manufacturing of nylon and other synthetic fibers. It is also used in slimicides as a disinfectant and antiseptic and in medicinal preparations such as mouthwash and sore throat lozenges	^70^
13	styrene	C_8_H_8_	104.15	909	0.5	2.7	+1.84	benzaldehyde	used in flavoring and perfume synthesis	^45,71^
14	acetovanillone	C_9_H_10_O_3_	166.17	1158	3.04	5–6	+1–1.5	vanillin	used in perfumes and fragrances, cleaning products, and candles, in the food industry to flavor chocolate, baked goods, and ice cream, and in medicines to mask unpleasant flavors	^72^
15	p-cresol	C_7_H_8_O	108.14	1030	240	8–11	+1.04	p-hydroxy benzaldehyde	intermediate used in medicine, perfume, and pesticides for the synthesis of drugs like hydroxyl ampicillin, trimethoprim, and 3-methoxy benzaldehyde	^73^
16	m-cresol	C_7_H_8_O	108.14	1030	23.5	5	+1.08	methyl-*p*-benzoquinone and 3-hydroxy benzaldehyde	used for the synthesis of antibacterial agents, antioxidants, polymer stabilizers, flavoring	^74^
17	o-cresol	C_7_H_8_O	108.14	1046	20	9–11	+1.04	salicylaldehyde	used in shampoos and food preservatives	^75^
18	1-ethyl-2-methyl-benzene	C_9_H_12_	120.19	866.5	0.74	3.3	+2.40	aceto phenone	fragrance in soaps and perfumes, flavoring agent in foods, solvent for plastics and resins.	^76^
19	1,2,3-trimethyl -benzene	C_9_H_12_	120.19	900	0.066		+2.23	trimethyl phenols	flavoring agent in food	^77^
20	1,2-dimethoxy-benzene	C_8_H_10_O_2_	138.16	1084	1.42	2.5	+2.37	polyveratrole	used as a conducting polymer, device technologies	^78^
21	3,4-dimethoxy toluene	C_9_H_12_O_2_	152.19	1051	1.01	3	+1.0	3,4-dimethoxybenzaldehyde and 3,4-dimethoxy benzoic acid	antimicrobial preservatives in food and beverages, especially in carbonated beverages.	^79^
22	2,6-xylenol	C_8_H_10_O	122.07	1000	6.18	5.1	+2.36	toluene	used in oil refining and the manufacturing of paints, lacquers, explosives (TNT), and glues	^80^
23	4-methyl guaiacol	C_8_H_10_O	138.16	1092	6.71	7	+0.43	vanillin	used in perfumes, fragrances, cleaning products, and candles, food industry to flavor chocolate, baked goods, ice cream, and medicines to mask unpleasant flavors.	^81^
24	mesitol	C_9_H_12_O	136.19	1046	1.58		+1.23	quinhydrone	used to measure the hydrogen ion concentration (pH) of a solution in a chemical experiment	^82^
25	*p*-xylenol	C_8_H_10_O	122.17	900	6.13	5.1	+1.10	2,5-dimethyl-1,4-benzoquinone/ortho-vanillin	used as a dienophile in Diels–Alder reactions/use in the study of mutagenesis and as a synthetic precursor for pharmaceuticals	^83^
27	propylguaiacol	C_10_H_14_O_2_	166.21	1000	0.89	4.8	+1.30	phenol	used in the manufacturing of nylon and other synthetic fibers. It is also used in slimicides as a disinfectant and antiseptic, and in medicinal preparations such as mouthwash and sore throat lozenges	^84^
28	3,4-xylenol	C_8_H_10_O	122.16	1138	5.45	7	+1.25	benzoquinone	it is used as a fungicide, as a reagent in photography, and to make dyes and other chemicals	^85^
29	pentamethyl phenol	C_11_H_16_O	150.22	968	0.20		+1.40	duroquinone	used as natural oxidants in various industrial chemical processes	^86^
30	tricresol	C_9_H_12_O	136.19	1072	1.88		+1.30	2,3,5-trimethyl-*p*-benzoquinone	used in the preparation of α-tocopherols (vitamin E)	^87^
31	isoeugenol	C_10_H_12_O_2_	164.20	1080	0.81	6.5	+0.96	vanillin	used in perfumes and fragrances, cleaning products, and candles, in the food industry to flavor chocolate, baked goods, and ice cream, and in medicines to mask unpleasant flavors	^88^
32	p-propyl anisole	C_10_H_14_O	150.21	941	0.066		+1.66	spiropyrrolidines and spirolactones	treatment of various diseases, including cancer, Alzheimer’s disease, and HIV spirolactones have diverse biological activities, including anticancer, antiviral, and antitumor properties	^30^

Other aromatic molecules in Table 1 (e.g., toluene, xylenes, cresols, and anisoles) exhibit oxidation potentials > 1 V, reflecting high reactivity under oxidation.^44^ Table 1 summarizes typical literature data, aiding in the prediction of reaction behavior and selectivity. Certain molecules (e.g., catechol and p-cresol) show good water solubility, influenced by pH, temperature, and depolymerization-induced hydrophilic groups.^45^ However, product selectivity hinges on oxidation methods used, enabling tailored synthesis for specific industrial needs.^46^

Aliphatic lignin-derived molecules (e.g., alkanes, alkenes, and alcohols) from lignin depolymerization (Table 2) exhibit distinct properties for industrial use. For instance, 2-methyl-1-butanol oxidizes readily to aldehydes/carboxylic acids, whereas longer-chain alcohols like 1-pentanol and 1-hexanol are less reactive due to steric hindrance. Understanding their oxidation potential is key to enhancing material stability, but current methods lack efficiency and selectivity. Further research is needed to unlock their full industrial potential. Table 3 lists key monolignols (*p*-coumaryl, coniferyl, and sinapyl alcohols) critical to lignin’s structure. Their oxidation via enzymatic, chemical, or electrochemical methods yields industrially relevant products like vanillin, syringaldehyde, and ferulic acid.^28,47,48^ Though sinapyl alcohol remains understudied. Other lignin derivatives (e.g., biphenyl and phenylethyl alcohol) oxidize to ketones/aldehydes for chemical applications or as polymer precursors (thermoplastics and resins).^49,50^ However, lignin-based polymer synthesis requires the optimization of methods and material properties for broader viability.

**Table 2:** Lignin-Derived Aliphatic Molecules: Properties, Oxidation Products, and Uses of Oxidative Products

s. no	common name	molecular formula	molecular weight g/mol	density Kg/m^3^ (298 K)	solubility g/L	pH	oxidation potential (V)	products	use of product	references
1	4-methyl-2-hexanol	C_7_H_16_O	116.20	807.5	819	7	+2.5	4-methyl-2-hexanone	used in paint, paint thinner, and to dissolve oils and waxes	^89^
2	Methyl-valerate	C_6_H_12_O_2_	*116.16*	875	5.06	12	+1.7	methyl pentanoate	used in fragrances, beauty care, soap, and laundry detergents	^90^
3	2-methyl-1-propanol	C_4_H_10_O	74.12	802	140		+1.5	2-methyl propanoic acid	used to manufacture esters for flavors and perfumes as a disinfecting agent.	^91^
4	2-methyl butanoate	C_6_H_12_O_2_	116.15	900	150	6.5	+0.50	1-butene	used to produce a wide variety of chemicals in the gasoline and rubber processing industry	^92^
5	2-methyl-1-butanol	C_5_H_12_O	88.15	815	31	6.8–7.2	+1.65	isovaleric acid	Widely used in perfumery	^93^
6	1-pentanol	C_5_H_12_O	*88.14*	811	27	6.4	+1.28	valeric acid	used as an intermediate in the manufacture of flavors, perfumes, ester-type lubricants, plasticisers, and vinyl stabilizers	^94^
7	1-hexanol	C_6_H_14_O	102.18	814	5.9	6	+0.85	caproic acid	used directly as feed additives, antimicrobials, plant growth promoters, lubricants, fragrances, paint additives, and pharmaceuticals	^95^
8	Dimethyl oxalate	C_4_H_6_O_4_	*118.09*	1148	60		+1.3	dimethyl carbonate	used to make coatings, adhesives, and cleaning agents	^96^
9	2-butanol	C_4_H_10_O	74.12	806	290	6–9	+0.65	butanone	used in glues, as a cleaning agent, paints, and other coatings	^97^

**Table 3:** Lignin-Derived Intermediate and Macromolecules: Properties, Oxidation Products, and Uses of Oxidative Products

s. no	common name	molecular formula	molecular weight g/mol	density Kg/m^3^ (298 K)	solubility (g/L)	pH	oxidation potential (V)	oxidation products	use of product	references
1	*p*-coumaryl alcohol	C_9_H_10_O	150.17	1201	6.97	8–11	+2.1	*p*-coumaric acid	used in the synthesis of fragrances and polymers	^47^
2	biphenyl	C_12_H_10_	154.21	1040	0.0045		+1.2	o-hydroxy biphenyl	used as an agricultural fungicide	^44,98^
3	2-phenoxy-1-phenylethanol	C_14_H_14_O_2_	214.26	1080	not soluble in water		+1.3	phenyl acetaldehyde	serves as a building block in organic synthesis, used in the polymer industry, flavoring agent	^28^
4	2-hydroxyacetophenone	C_8_H_8_O_2_	136.15	1170	5.5	9.5	+0.90	2-phenoxy acetophenone	used in manufacturing of pharmaceuticals, polymerization initiator, dyes and pigments	^28^
5	phenylethyl alcohol	C_8_H_10_O	122.16	1015	1.3		+0.75	2-phenoxy-1-phenylethane	used as a chemical intermediate, in the production of polymers and preparation of surfactants, pesticides, resins	^28^

## Global Demand for the Possible Lignin-Derived Products

The global demand for aromatic compounds, such as vanillin, guaiacol, syringaldehyde, and catechol products, that can be derived from the electrooxidation of lignin continues to grow across sectors, including food flavoring, pharmaceuticals, agrochemicals, and polymer industries (Table 4). Currently, most of these compounds are synthetically produced from fossil-derived precursors like benzene, toluene, and phenol through energy-intensive and environmentally taxing processes.^51,52^ For instance, vanillin, with a global demand exceeding 20,000 tons annually, is predominantly manufactured from petrochemical guaiacol,^53^ while guaiacol itself is widely used as an intermediate in the synthesis of pharmaceuticals (e.g., expectorants), agrochemicals, and fragrances. Similarly, catechol and syringaldehyde are used in the production of antioxidants, adhesives, polymers, and synthetic flavors.^54^ The projected growth in these markets, driven by rising consumer demand for biobased alternatives and regulatory pressures toward sustainable sourcing, presents a significant opportunity for lignin-based production. Electrochemical valorization of lignin offers a renewable, carbon-efficient pathway to meet this demand, reducing reliance on petroleum feedstocks and aligning with global efforts toward green chemistry and circular bioeconomies.^55^

**Table 4:** Market Overview of the Possible E-LignoX Products and Their Current Industrial Profiles

product	global demand	production volume	market price (USD/ton)	current source	references
vanillin	∼16,000–20,000 tons	∼18,000 tons	$11,000–$18,000	guaiacol (petrochemical)	^55,99^
syringaldehyde	Niche	<5000 tons (est.)	$3000–$7000	lignin derivatives	^100–102^
guaiacol	∼10,000–15,000 tons	∼12,000 tons	$2000–$5000	petrochemical guaiacol	^103^
catechol	>100,000 tons	∼110,000 tons	$2500–$4000	phenol/benzene	^104^
phenol	>10 million tons	∼11 million tons	$1000–$1500	crude oil (cumene process)	^105,106^
benzaldehyde	∼50,000–60,000 tons	∼60,000 tons	$2000–$4000	toluene oxidation	^107,108^
vanillic acid	Niche	<5000 tons	$6000–$10,000	from vanillin	^109^
ferulic acid	∼3000–5000 tons	∼4000 tons	$800–$1200	biomass or synthetic	^110^

## Electrocatalytic Oxidation

In recent decades, electrochemical techniques have gained traction in biomass research, focusing on two approaches: untargeted lignin degradation and targeted degradation for specific product formation.^111^ Targeted degradation is more complex and has received less attention.^112^ Bailey and Brooks pioneered untargeted electrochemical lignin depolymerization in the 1940s, using mercury/lead electrodes in 1% NaOH at 1–3 A/dm^2^ on organosolv lignin, yielding complex mixtures after 12–96 h.^113^ Though simple, such methods produce intricate product blends requiring advanced analytical separation.^114^ Research on electrochemical depolymerization of lignin stagnated post-1940s but resurged in the last 20–30 years, with Stephenson et al. reviewing lignin oxidation via enzymatic, homogeneous, and heterogeneous catalysis.^40,144,161^ However, the persistent challenge of complex product mixtures has shifted focus toward targeted lignin depolymerization to avoid intricate purification processes. Recent studies highlight E-LignoX as a promising, energy-efficient technology for targeted lignin depolymerization, despite its early stage development.^115–119^

E-LignoX offers sustainability, environmental friendliness, high selectivity, and precise control over lignin degradation while simultaneously producing hydrogen and oxygen. By optimizing the electrode voltage, composition, and current density, the final products can be achieved with exceptional selectivity. E-LignoX can proceed through direct and indirect pathways, as shown in Figure 3.

**Figure 3:**
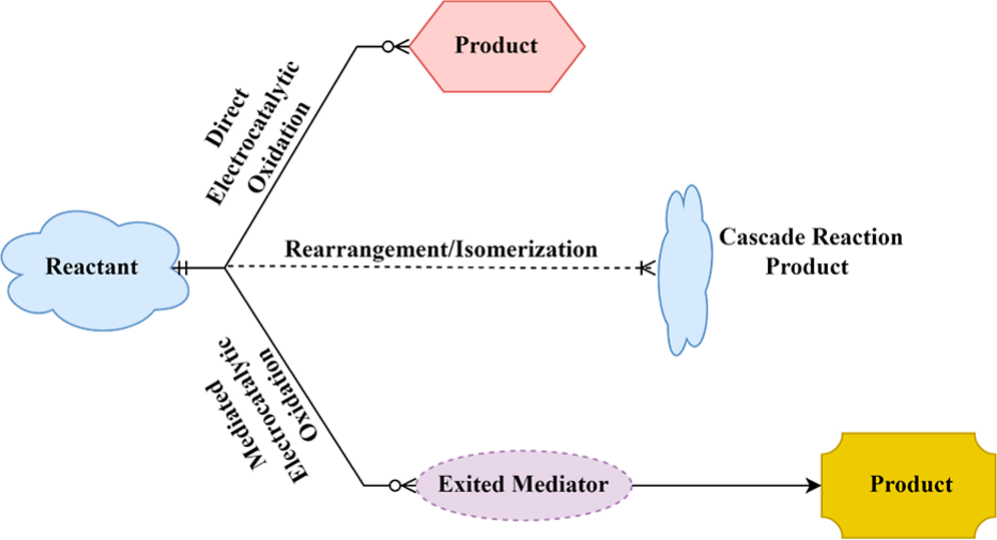
General reaction mechanisms of electrocatalytic oxidation for organic transformations.

## Direct Electrocatalytic Oxidation

Direct E-LignoX is a highly promising strategy in which electrons are directly transferred from lignin to solid electrocatalysts immobilized on the electrode surface, typically under a low applied potential. The oxidation mechanism begins with the generation of hydroxyl radicals from water or hydroxide ions in the electrolyte. Through chemisorption on the anode surface, these radicals lead the electrocatalyst to a higher oxidation state, forming catalytic species that efficiently oxidize organic molecules.^120–122^ The complex sequence of reactions can be summarized as follows

1 M+H2O→M(OH·)ads+e−+H+

2 M(OH·)ads→MO+e−+H+

3 $$M{\left(R\right)}_{ads}+MO\to RO+2M$$

Moreover, oxygen evolution reaction (OER) from water oxidation can occur at the anode, competing with the previous reactions

4 M(OH·)ads+M(OH·)ads→O2+2M+2e−+2H+

5 $$MO+MO\to O_{2}+2M$$

Additionally, another oxidation mechanism involving the generation of reactive oxygen species, including hydrogen peroxide and its oxidation products, such as the superoxide ion, can occur at the anode. Direct ECO pathways close the catalytic cycle when applied potentials (>1.6 V vs RHE) oxidize lignin phenolic units to surface-bound radical cations. These radicals cleave β–O–4 bonds and subsequently generate product molecules, while water or hydroxide fills the electron vacancy to regenerate the catalyst.^39^ Tuning the potential and selecting materials (e.g., Ni- or Co-doped oxides) maximizes lignin oxidation selectivity over OER.^123^ The main limitations of lignin electro-oxidation are the need to operate in a highly basic environment, typically with NaOH solutions up to 3 M, often at temperatures above ambient temperature, and the formation of complex product mixtures, making separation and recovery difficult. To address this last issue while steering the process toward the selective production of valuable chemical building blocks, research has shifted its focus to the targeted electrooxidation of lignin.

The earliest electrooxidation studies on raw lignin investigated the activity of transition-metal-based anodes, producing commercially valuable compounds such as vanillin, guaiacol, syringaldehyde, and other aromatic derivatives, though with low selectivity. Tolba et al. investigated the electrooxidation of Kraft lignin using four different IrO_2_-based electrodes (Ti/SnO_2_–IrO_2_, Ti/RuO_2_–IrO_2_, Ti/Ta_2_O_5_–IrO_2_, and Ti/TiO_2_–IrO_2_), identifying vanillin and vanillic acid as the primary oxidation products. The kinetics of lignin degradation was analyzed using in situ UV–vis spectroscopy, revealing Ti/RuO_2_–IrO_2_ as the most promising electrocatalyst, demonstrating the highest stability and activity for lignin degradation.^124^ Through a redox electrocatalytic system with Cu electrodes (cathode) and Pb/PbO2 electrodes (anode) in a sodium hydroxide solution, Liu et al. conducted the electrochemical degradation of bamboo lignin. Analysis using GC–MS identified 24 products, including vanillin, syringaldehyde, and *p*-coumaric acid, obtained in high yields. Furthermore, by investigating the effects of operational parameters such as lignin concentration, current density, and temperature, they determined the optimal conditions for achieving the highest yields of vanillin, syringaldehyde, and *p*-coumaric acid.^120^

Another study on lignin depolymerization and the impact on the OER was conducted by developing Ni–Sn alloys with varying Ni/Sn ratios through coelectrodeposition. The results showed that higher Ni/Sn ratios enhanced OER performance, whereas at lower cell voltages, lignin depolymerization was more prominent with a net decrease of OER faradaic efficiency.^125^ Using a nickel foam electrode under alkaline conditions, Yan and colleagues depolymerized three ethanol organosolv lignins, achieving a maximum yield of 17.5% vanillin and syringaldehyde.^126^ Beyond the low selectivity, the main limitations include challenging product separation and catalyst deactivation. To the best of our knowledge, Parpot and co-workers achieved the most significant selectivity to oxidation products through the direct electrooxidation of raw lignin. Using PbO_2_ as the anode material, the electrooxidation of Kraft lignin achieved a vanillin yield of 64%.^62^ In the same study, nickel-based and dimensionally stable anodes, incorporating electroactive metal oxides on titanium, delivered comparable yields ranging from 56% to 63%. Promising outcomes are also achieved through the direct electro-oxidation of lignin-derived molecules. Qi et al. presented an interesting study on an efficient electrochemical strategy for the selective oxidation of 2-phenoxy-1-phenyle-ethanol, a dimer representative of Cα–Cβ bonds present in lignin, yielding aromatic monomers, through the modulation of the electronic structure of phosphorus (P)-doped CoMoO4spinels. The approach achieved 99% lignin derivative conversion and a maximum selectivity of 56% to benzoic acid, while potential tuning enabled the simultaneous hydrogenation of furfural. The introduction of phosphorus increased the level of antibonding, favoring the adsorption of Cα–Cβ bonds in lignin model compounds and thus enhancing the bifunctional electrocatalytic activity of the active site. In this work, they demonstrated the potential of spinels as bifunctionalel ectrocatalysts, facilitating both the oxidative cleavage of lignin and the reduction of small organic molecules for the synthesis of high-value chemicals through the modulation of the P anion.^127^ Moreover, the electrocatalytic oxidation of Cα-Cβ bonds was also explored by Wang et al., who demonstrated how Pt atoms anchored on nitrogen-doped carbon nanotubes (Pt/N-CNTs) allowed for achievement of 99% of 2-phenoxy-1-phenylethanol conversion and an excellent yield of 81% to benzaldehyde.^128^ Despite these advancements in the selective oxidation of lignin dimers, the issue of their limited solubility and consequently the solubility of lignin remains a significant challenge. Ionic liquids (ILs) have gained significant attention due to their exceptional ability to solvate lignin and its derivatives. In fact, ILs possess several beneficial properties, including a broad electrochemical window, low volatility, high thermal stability, and good miscibility with both organic and inorganic substances. Li et al. proposed the use of free-metal carbon electrodes for the electrocatalytic cleavage of C–C and C–O bonds in lignin model compounds dissolved in ILs. Under optimal conditions, the production of aldehydes and quinones can reach 90% yield, with a conversion rate surpassing 90%. Mechanistic studies indicate that carbon catalysts with a high density of surface defects enhance electron transfer during the oxidative cleavage of C–C bonds.^128^ Although progress has been made, this field still encounters challenges, including low product yields, limited selectivity, and dependence on noble metals.

The selective electro-oxidation of lignin-derived monomers, such as phenols and cresols, is still an underexplored field with limited mechanistic insights and challenges related to reproducibility. Phenolic compounds, often produced in pesticide and chemical manufacturing, are commonly viewed as environmental pollutants, which has led most research to concentrate on their complete electrocatalytic oxidation to CO_2_ rather than their selective valorization.^129,130^ TiO_2_-based and boron-doped diamond (BDD) anodes, widely used for phenol mineralization, offer valuable insights.^131^ For instance, TiO_2_ nanostructures demonstrate high oxygen evolution overpotentials, minimizing energy loss; however, their limited adsorption sites tend to favor partial oxidation products like hydroquinone and benzoquinone.^132^ Doping or the integration of 3D architectures, such as TiO_2_/activated carbon fibers, can enhance the adsorption and oxidation of intermediates.^133^ BDD electrodes, while effective for complete phenol mineralization at high current densities, tend to produce partial oxidation products (e.g., catechol) under lower current conditions. The surface oxygen functionalities, influenced by doping and operational parameters, are crucial in determining product selectivity.^131^

Ongoing experiments in the frame of the EPOCH project demonstrate that commercial BDD anodes in the m-cresol oxidation carried out in a flow cell resulted in rapid deactivation due to polymer formation, although activity was recovered through anodic polarization (>2.3 V vs SHE). More promising outcomes were observed with graphene/boron-doped mesoporous carbon anodes, achieving over 60% m-cresol conversion with 30% selectivity toward methyl-*p*-benzoquinone. Current research is focused on correlating catalyst properties, such as conductivity, heteroatom doping, and surface area, with the nature of oxidizing species (e.g., •OH, O^–^, or metal-oxo intermediates) to guide the rational design of more efficient catalysts.^134^ Future work should prioritize mechanistic studies to understand the role of surface oxygen species as well as material innovations such as heteroatom-doped carbons and mixed-metal oxides. Optimizing operational parameters, including pH, potential, and concentration, is key to minimizing polymerization, while standardized testing protocols under industrially relevant conditions, such as high concentrations and continuous flow, are necessary. Bridging these gaps will advance selective electro-oxidation toward scalable lignin valorization, a critical step in sustainable biorefinery systems.

## Indirect or Mediated Electrocatalytic Oxidation

Indirect oxidation reactions use an electrolyte as the reaction medium with an electrocatalyst or mediator aiding electron transfer between the electrode and lignin. This process includes heterogeneous electron transfer and homogeneous redox reactions. Mediators react with substrates and regenerate at the electrode, requiring stability in oxidized and reduced states without cleavage. Two main systems are used: laccase mediators (mediators dissolved in the electrolyte) and electrolytic mediators (those anchored to the electrode) (Figure 4).^135^ Electrolytic systems are environmentally friendly, as they eliminate the need for mediator recovery by ensuring surface stability. Detailed discussions follow on these systems.

**Figure 4:**
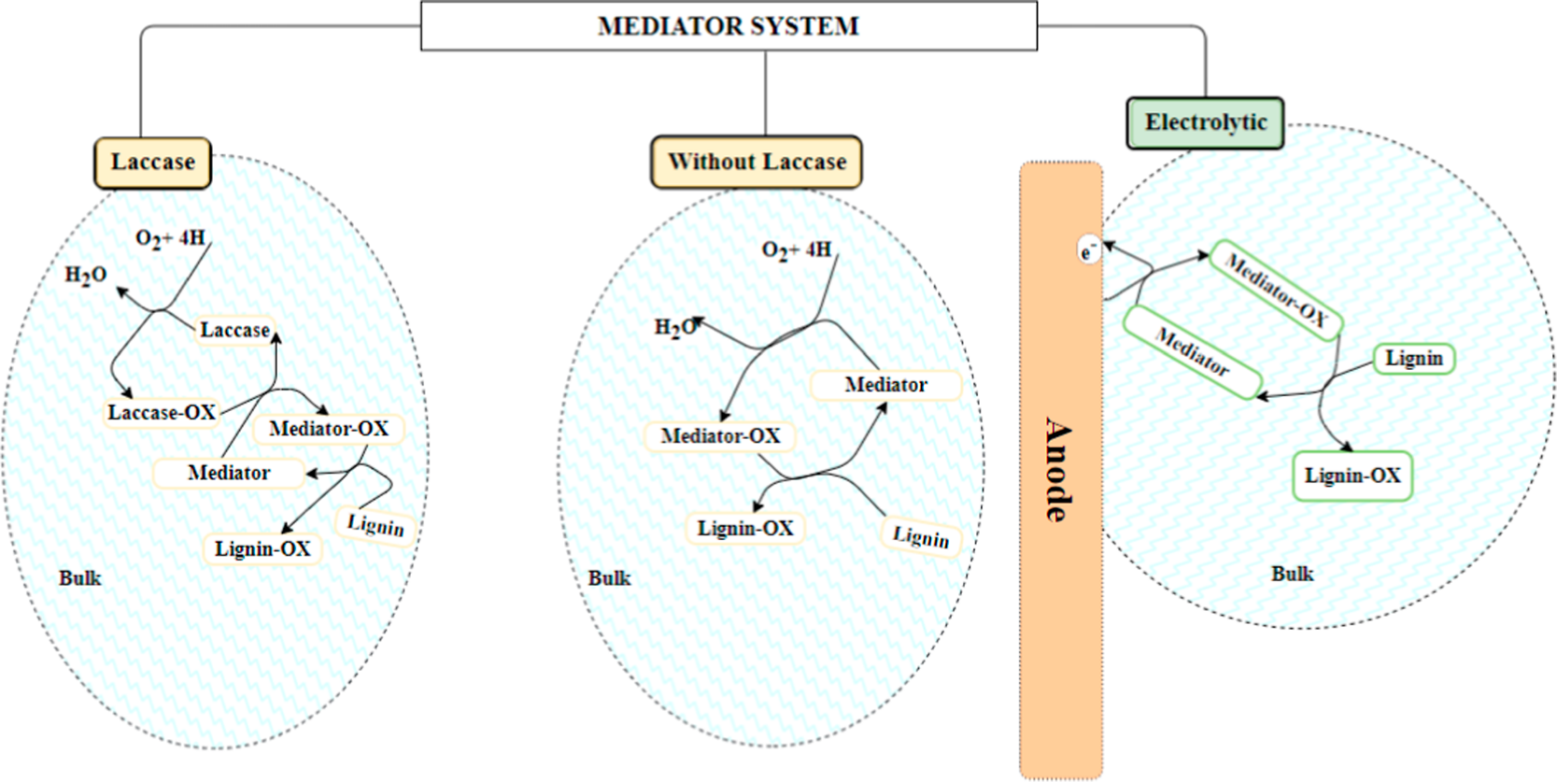
Laccase and electrolytic mediator system for enhanced electrooxidation. Adapted with permission from ref 136 Copyright 2013 Elsevier.

### Laccase-Mediator System (LMS)

The LMS combines laccase (an enzyme that oxidizes lignin) and a redox mediator. It oxidizes recalcitrant substrates via electron transfer,^137^ while mediators enhance this process and enable the enzyme’s continuous regeneration.^46^ ABTS’s discovery (1990) broadened LMS applications from lignin degradation to fine chemicals, bioremediation, and pollutant breakdown,^138^ enhancing industrial viability through improved selectivity/efficiency.^139^ Mediators like 2,2′-azino-bis­(3-ethylbenzothiazoline-6-sulfonic acid) (ABTS) and remazol blue enable oxidation of nonphenolic lignin (e.g., *Trametes hirsuta* laccase produces veratraldehyde/benzaldehyde from dimers),^140^ while inorganic mediators (nitrobenzene, polyoxometalates) also drive lignin oxidation.^26^ HBT’s enzymatic pathway yields Cα-carbonyl products, while mixed cleavage products are formed through an electrochemical pathway.^28^ Despite progress, lignin’s structural complexity challenges rational mediator design. Current research prioritizes stability, selectivity, and cost-effectiveness for scalable lignin valorization.^141–143^

Recent research highlights NHPI as the most effective mediator for the E-LignoX, particularly targeting β–O–4 linkages.^46,121^ It catalyzes organic oxidation under mild conditions using molecular oxygen and metal salts, generating the potent oxidant phthalimide-N-oxyl (PINO). Electron-donating groups on NHPI enhance the oxidation yields. The mechanism involves anodic oxidation of NHPI to PINO via proton-coupled electron transfer (Figure 5).^26^ PINO reacts with the substrate, regenerates NHPI via hydrogen atom transfer (the rate-determining step), and forms α-carbonyl products through further oxidation. This highlights NHPI’s efficiency and selectivity in lignin degradation. An efficient redox mediator must sustain multiple catalytic cycles.^46^ Transition metal complexes [e.g., potassium octocyanomolybdate, Fe­(II)] and organic laccase substrates (ABTS and TEMPO) exhibit high redox potential and stability at low concentrations but face cost and environmental constraints. Laccase mediators are categorized by the following mechanisms: HAT (NHPI, HBT, and VLA), ionic (TEMPO), and electron transfer (ABTS). HAT/ionic pathways yield Cα-carbonyl products, while electron transfer enables Cα-Cβ cleavage to phenolic monomers.^26,46,136^

**Figure 5:**
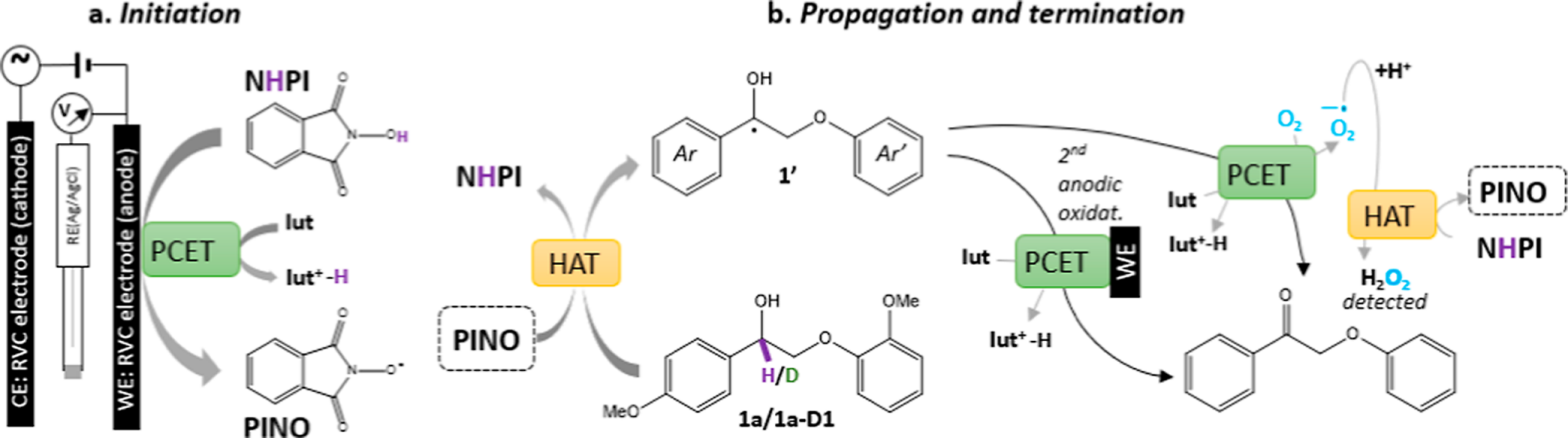
Mechanism of NHPI as a mediator in the electrolytic mediator system. Adapted with permission from ref 144 Copyright 2021 ACS.

### Mediated Oxidation without Laccase

Mediators can directly drive the electro-oxidation of lignin-derived molecules without requiring a laccase enzyme. For example, Rafiee et al.^145^ achieved chemoselective oxidation of primary alcohols in lignin to carboxylic acids using 4-acetamido-TEMPO (ACT) under mildly basic conditions, successfully applying this method to lignin from poplar wood chips via mild acidolysis. TEMPO and its derivatives are redox-active nitroxide radicals with two transitions, TEMPO/TEMPO^+^ (0.73 V vs NHE) and TEMPOH/TEMPO (−0.2 to −0.9 V vs NHE, pH-dependent), where TEMPO^+^ oxidizes alcohols to aldehydes, ketones, and acids.^146^ Semmelhack et al.^147^ pioneered this TEMPO-catalyzed oxidation, noting that in basic conditions, deprotonated alcohols bind to TEMPO^+^ followed by hydride transfer. At the same time, in acidic media, TEMPO^+^ directly abstracts a hydride. Additionally, imidoxyl radicals like PINO efficiently abstract hydrogen from weak C–H bonds, catalyzing the oxidation of alcohols, alkenes, and other C–H bonds,^146,148^ with NHPI forming via PINO hydrogen abstraction (Figure 6).

**Figure 6:**
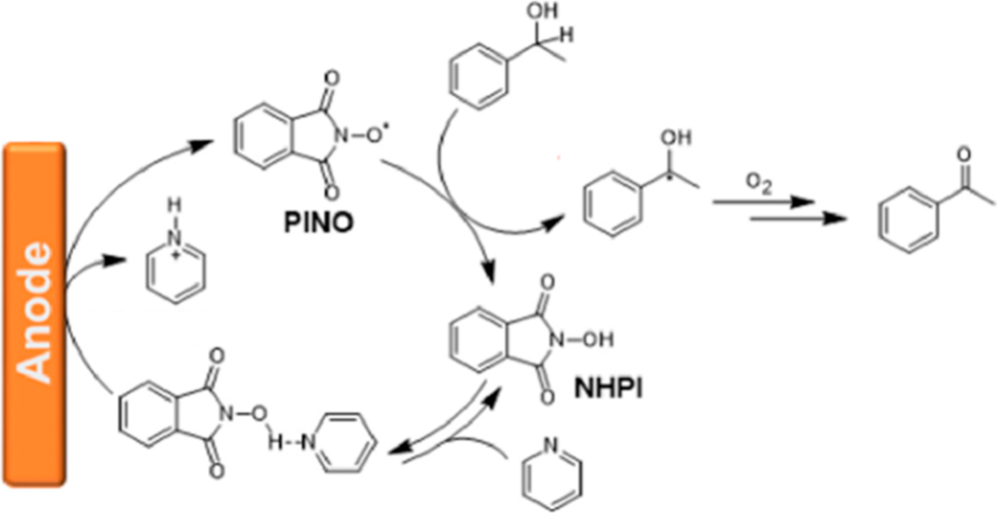
Catalytic cycle of N-hydroxyphthalimide (NHPI)-mediated alcohol electrooxidation with PINO-mediated electrochemical oxidation of lignin derivatives. Adapted with permission from ref 144 Copyright 2021 ACS.

### Electrolytic Mediator Systems

The EMS system facilitates electron exchange between the substrate and electrode, offering advantages over LMS, such as broader pH and temperature ranges and the use of high-redox-potential mediators.^149,150^ While mediators like NHPI, VLA, HBT, ABTS, and tris­(4-bromophenyl)­amine have shown promise in oxidizing nonphenolic β–O–4 dimers in EMS, results differ from LMS. For instance, HBT in LMS yields only Cα-carbonyl products, whereas in EMS, it produces a mix of Cα-carbonyl and Cα-Cβ cleavage products.^46^ Sannami et al.^148^ demonstrated EMS oxidation of a nonphenolic β-O-4 lignin model using TEMPO or AcNH-TEMPO, with electrolyte choice significantly affecting chemoselectivity: LiClO_4_/CH_3_CN–H_2_O favored Cα-carbonylation, while a dioxane-carbonate buffer achieved high Cγ-carboxylation yields (72–93%). Despite these advances, systematic studies comparing laccase mediators in EMS are lacking, and challenges remain in achieving selective and efficient oxidation of nonphenolic lignin. Further research is needed to optimize EMS for lignin valorization, although its versatility holds the potential for enhancing electrochemical reactions in various applications.

An alternative approach in mediated electro-oxidation involves hybrid catalytic-electrocatalytic systems, where electrodes generate oxidants like H_2_O_2_ in situ, paired with solid catalysts (e.g., TS-1) to drive selective oxidation.^151^ This integrated setup minimizes H_2_O_2_ decomposition by directly coupling its generation with catalytic conversion, often through a porous catalyst layer on the anode. Despite its potential to enhance efficiency and selectivity, this method is unexplored in the literature, highlighting a critical research gap. Another emerging strategy employs microbial electrodes, where enzymes or microorganisms are immobilized on electrodes to enable direct electron transfer, bypassing energy-intensive cofactors like adenosine triphosphate or nicotinamide adenine dinucleotide.^152,153^ While promising for lignin valorization, these systems face challenges due to their sensitivity to impurities in biobased feedstocks, which limits practical scalability.

### Direct vs Mediated E-LignoX, Determinants of Product Selectivity

The direct E-LignoX and mediated E-LignoX routes lead to different products with distinct selectivity. In direct E-LignoX, the organic compound is oxidized directly at the anode, and the selectivity of the product is determined by the reaction pathway and intermediate species formed. The selectivity of the product can be influenced by factors including the choice of electrode material, the nature and concentration of the electrolyte, the current density and potential applied, and the presence of other reactants or intermediates. Direct E-LignoX provides high selectivity and yield for certain reactions, especially when the reaction conditions are carefully controlled. In mediated E-LignoX, a mediator facilitates the oxidation of an organic compound. The mediator affects the reaction pathway and intermediate species formed during oxidation, leading to different product distributions. Mediated E-LignoX may achieve a higher selectivity and yield for certain reactions.

When it comes to the direct E-LignoX, the inherent complexity and size of the lignin polymer pose a substantial obstacle. Specifically, the restricted accessibility of lignin linkages by active anode sites can hinder the selectivity of certain desired products. Therefore, a mediator system can serve as a viable alternative to address these issues. Mediators are increasingly employed in electrocatalysis to facilitate the transport of redox electrons to the target redox site to overcome the existing issues. The judicious use of mediators allows for the effective contact of lignin polymers with electrochemically activated species, which could lead to improved activity and selectivity. Mediated E-LignoX is particularly useful when simultaneous hydrogen gas generation is desired, as it helps to avoid undesirable side reactions by consuming protons, electrons, and ions for activation. The choice between direct and mediated ECO routes depends on several factors, including the nature of the organic molecule to be oxidized, the desired product, the reaction conditions, and the available resources. Direct ECO can be more straightforward and cost-effective for simple organic compounds, while mediated ECO can provide higher selectivity and yield for complex organic compounds.^143^ One significant limitation lies in the potential instability or degradation of mediators under prolonged electrochemical conditions, which can affect the long-term performance and scalability. Additionally, the redox potential of mediators must be carefully matched with those of the substrate and catalyst to avoid unwanted side reactions or inefficiencies. The synthesis and separation of mediators, especially in complex systems, may also introduce cost and operational complexity.^140^ Addressing these issues is crucial for the practical deployment of mediated electrocatalysis in industrial settings.

Therefore, the selection of direct and mediated routes on product selectivity in E-LignoX reactions is a complex interplay of several factors, including the nature of the organic compound, the reaction conditions, and the choice of mediator or catalyst. As a result, selecting the most appropriate E-LignoX route requires careful consideration of these factors to achieve the optimal selectivity and yield. The most optimal pathway for electrocatalytic oxidation can be devised through an extensive review of the existing literature, considering the initial molecules’ inherent characteristics, as illustrated in Figure 7. Recent life cycle assessment and techno-economic analyses have shown that E-LignoX can reduce greenhouse gas emissions by up to 40% and lower energy consumption by approximately 30%, primarily due to their operation under milder conditions (ambient to ∼80 °C, atmospheric pressure) and their compatibility with renewable electricity sources.^154^ In contrast, thermocatalytic processes typically require elevated temperatures and pressures, resulting in higher energy use and carbon intensity.^155,156^ E-LignoX offers additional advantages through integration with hydrogen evolution reactions (HER), enabling simultaneous production of green hydrogen and high-value chemicals, thereby enhancing sustainability and reducing operational costs.^156,157,158^ For example, Zhang et al.^30^ demonstrated that coupling lignin oxidation at a Ni–Fe oxyhydroxide anode with HER at a Pt cathode yields 18 wt % vanillin and over 95% Faradaic efficiency for H_2_, with a cell voltage approximately 0.5 V lower than conventional water electrolysis. Similarly, Moges et al.^164^ discussed lignin-assisted electrolysis using transition-metal-based anodes, which can simultaneously depolymerize organosolv lignin into aromatic monomers (∼22 wt%) and generate high-purity hydrogen, while lowering energy consumption by up to ∼40% compared to a standalone HER system. These examples underscore the potential of VAARs–HER integration to enhance both energy efficiency and economic viability by generating dual revenue streams. Furthermore, emerging studies highlight the promise of cathodic electrochemical reduction of lignin or its intermediates to produce valuable aliphatic compounds, alcohols, and hydrocarbons.^159–161^ Although less explored than anodic pathways, such reductive strategies offer new avenues for lignin valorization and warrant further investigation, particularly as part of an integrated and energy-efficient biorefinery framework.

**Figure 7:**
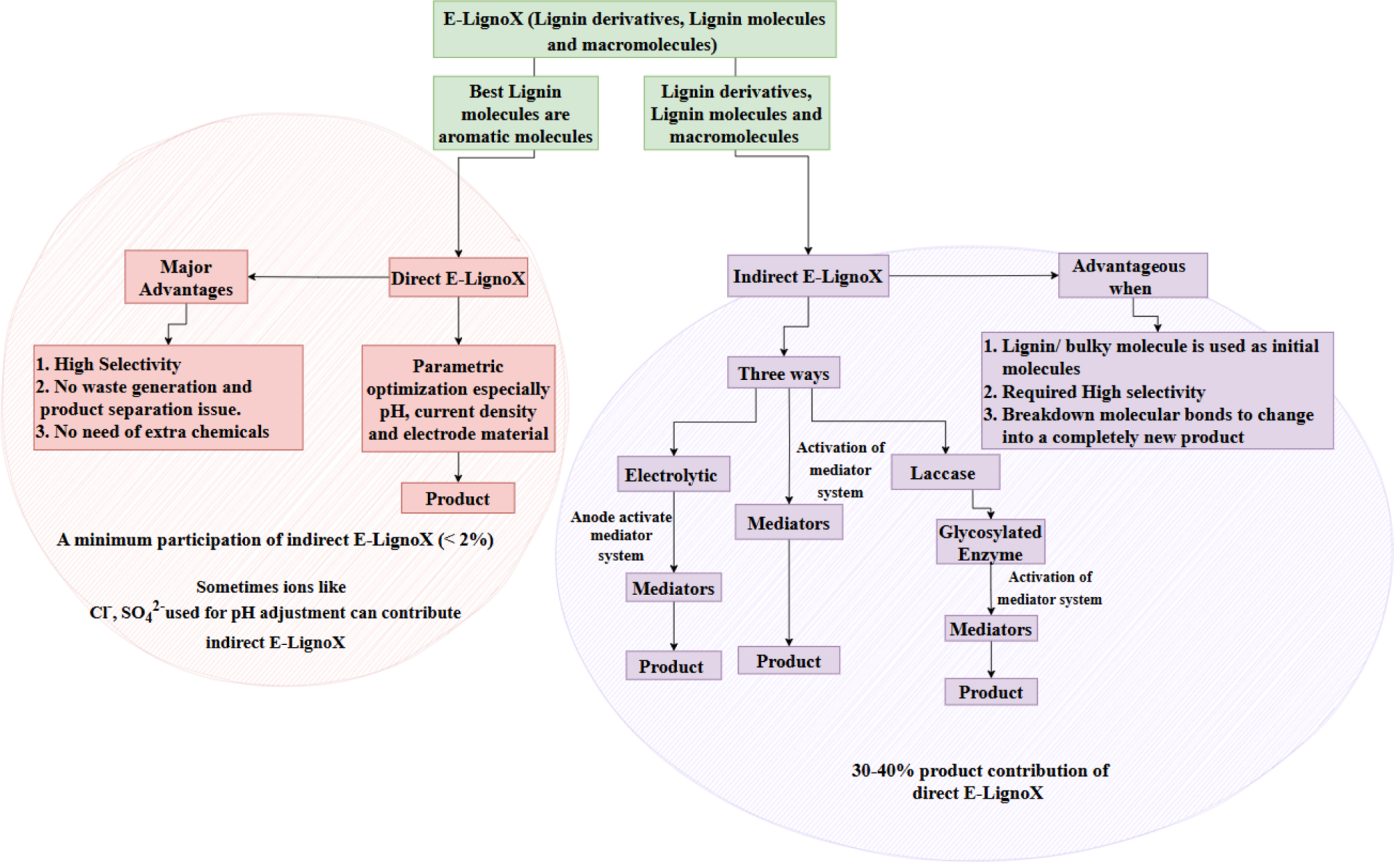
Electrocatalytic pathways for selective oxidation of lignin-derived compounds.

## Conclusions

This perspective presents a comprehensive overview of E-LignoX, highlighting its potential to convert lignin-derived molecules into high-value industrial chemicals. While the existing literature emphasizes the industrial promise of these molecules, critical gaps persist in understanding oxidation pathways under E-LignoX conditions, particularly those governing high-selectivity product formation. Addressing these gaps offers a strategic opportunity to advance mechanistic insights and optimize target product yields.

Recent advances in E-LignoX strategies are critically analyzed, with a focus on two dominant approaches: direct electrode–surface oxidation and mediator-assisted oxidation. Direct E-LignoX, while cost-effective for simple substrates, struggles with lignin’s structural complexity.^162^ Bulky substituents and steric hindrance at electrode–electrolyte interfaces frequently impede substrate–catalyst interactions, diminishing the selectivity. Conversely, mediator-assisted E-LignoX overcomes these limitations by transporting redox electrons to obstructed reaction sites, boosting both the catalytic activity and selectivity. Mediators such as TEMPO or metal complexes enable precise oxidation of targeted lignin linkages (e.g., β-O-4 bonds), steering product distributions toward desirable chemicals.

E-LignoX efficiency is further governed by molecular-level factors, including the electronic and steric properties of lignin-derived intermediates, electrocatalyst design, and interfacial reaction kinetics. Strategic parameter optimization, such as tailoring electrode materials (e.g., Pt, carbon-based catalysts) or adjusting electrolyte pH, can suppress side reactions and enhance selectivity. Furthermore, E-LignoX aligns with sustainability goals by employing renewable electricity to drive chemical transformations, upgrading low-value lignin streams into value-added products with reduced environmental footprints.

Despite its potential, E-LignoX remains in an early development stage. Several key challenges in scaling up electrocatalytic lignin valorization include the need for continuous-flow reactor designs that ensure uniform lignin distribution and adequate mass transport, as well as the development of fouling-resistant anode materials to maintain long-term activity. Improving energy efficiency, ideally by coupling lignin oxidation with hydrogen evolution to lower overall cell voltage, will be critical for economic viability.^163,164^ For downstream processing, we propose integrating in-line separation techniques, such as reactive solvent extraction or membrane-based fractionation, directly with the electrochemical reactor to enable continuous product recovery at high purity. Embedding these ECO units within the existing biorefinery infrastructures can leverage on-site lignin streams, shared utilities, and coproduction of residual oligomers for materials or process heat, thereby enhancing the overall process economics and sustainability. To further accelerate progress in E-LignoX technologies, interdisciplinary collaboration will be essential. The integration of computational chemistry can guide rational catalyst design and mechanistic understanding, while machine learning and AI offer tools for optimizing reaction conditions and predicting product selectivity. Moreover, advances in synthetic biology and bioengineering can enable the development of tailored lignin feedstocks through plant modification or enzyme engineering. By leveraging these complementary approaches, the field can move closer to realizing efficient, scalable, and sustainable lignin valorization pathways.

## ∥

Ordos Laboratory, 6 Huineng Building, Kangbashi, Inner Mongolia, China

## List of Abbreviations

ABTS: 2,2′-azino-bis-(3-ethylbenzothiazoline-6-sulfonic acid)
ACT: 4-acetamido-TEMPO
ATP: adenosine triphosphate
E-LignoX: electrocatalytic lignin oxidation
EMS: electrolytic mediator system
FE: Faradaic efficiency
HAT: hydrogen atom transfer
HBT: 1-hydroxybenzotriazole
LDH: layered double hydroxides
LMS: laccase mediator system
NADH: nicotinammina adenina dinucleotide
NF: (carbon) nanofibres
NHPI: N-hydroxyphthalimide
PINO: phthalimide-N-oxyl
TEMPO: 2,6,6-tetramethyl-1-piperidine N-oxyl
VLA: violuric acid
